# Supplementary material for: Invasive Burmese pythons alter host use and virus infection in the vector of a zoonotic virus
Source: Commun Biol. 2021 Jun 28;4:804. doi: 10.1038/s42003-021-02347-z (PMC8239020; doi:10.1038/s42003-021-02347-z)
Supplement: Supplementary file 2 — Supplementary Information [file 42003_2021_2347_MOESM2_ESM.pdf]

Supplementary Table 1. Model parameter estimates (β), SE's, 95% Confidence intervals (CI), z-values and p-values of models used to explain the variation in relative cotton rat host use and Everglades virus infection rate in vector mosquitoes.

Models and parameters	*β*	SE	Z-value	*p*	Lower CI	Upper CI
**Relative Cotton rat host use**						
Cotton rat activity + Non-rodent diversity						
Cotton rat activity	0.17	0.07	2.37	0.018	0.03	0.31
Non-rodent diversity	-1.20	0.36	-3.30	0.001	-1.91	-0.49
Non-rodent activity						
Non-rodent activity	-10.33	7.36	-1.40	0.160	-24.67	4.01
Cotton rat activity						
Cotton rat activity	0.25	0.12	1.99	0.046	0.01	0.49
Non-rodent activity + Non-rodent diversity						
Non-rodent activity	0.51	5.77	0.09	0.929	-10.74	11.76
Non-rodent diversity	-1.52	0.54	-2.80	0.005	-2.58	-0.46
Cotton rat activity + Non-rodent activity						
Cotton rat activity	0.22	0.12	1.88	0.060	-0.01	0.45
Non-rodent activity	-7.86	6.23	-1.26	0.207	-20.00	4.29
Non-rodent diversity						
Non-rodent diversity	-1.786	0.536	-3.33	0.001	-2.83	-0.74
**EVEV Infection rate**						
Cotton rat activity + Relative Cotton rat host use						
Cotton rat activity	-0.19	0.11	-1.74	0.082	-0.40	0.02
Relative Cotton rat host use	4.06	1.25	3.24	0.001	1.61	6.50
Relative Cotton rat host use						
Relative Cotton rat host use	2.95	1.37	2.16	0.031	0.29	5.61
Cotton rat activity + Relative Cotton rat host use + Non-rodent diversity						
Cotton rat activity	-0.19	0.11	-1.71	0.087	-0.41	0.03
Relative Cotton rat host use	3.99	1.44	2.78	0.005	1.19	6.79
Non-rodent diversity	-0.59	5.88	-0.10	0.921	-12.06	10.89
Non-rodent activity						
Non-rodent activity	-10.39	8.05	-1.29	0.197	-26.10	5.31
Non-rodent diversity						
Non-rodent diversity	-0.79	0.54	-1.46	0.144	-1.84	0.26
Non-rodent activity + Relative Cotton rat host use						
Non-rodent activity	-3.00	7.46	-0.40	0.687	-17.55	11.54
Relative Cotton rat host use	2.59	1.71	1.52	0.129	-0.74	5.92
Non-rodent activity + Non-rodent diversity						
Non-rodent activity	-6.22	8.56	-0.727	0.47	-22.90	10.46
Non-rodent diversity	-0.54	0.65	-0.828	0.41	-1.80	0.73
Cotton rat activity + Non-rodent activity						
Cotton rat activity	-0.08	0.15	-0.57	0.566	-0.39	0.21
Non-rodent activity	-11.50	8.38	-1.37	0.170	-27.84	4.84
Cotton rat activity + Non-rodent diversity						
Cotton rat activity	-0.08	0.13	-0.57	0.569	-0.33	0.18
Non-rodent diversity	-0.85	0.54	-1.57	0.117	-1.92	0.21
Cotton rat activity						
Cotton rat activity	-0.03	0.15	-0.20	0.841	-0.31	0.26

Supplementary Table 2. Pearson's correlation matrix model variables.

	Cotton Rat Activity	Non-rodent Activity	Cotton Rat : Other Animal Blood Meals	Non-rodent Diversity	Proportion EVEV Positive Pools
Cotton Rat Activity	1	-0.144	0.522	-0.189	-0.109
Non-rodent Activity	-0.144	1	-0.513	0.555	-0.458
Cotton Rat : Other Animal Blood Meals	0.522	-0.513	1	-0.797	0.566
Non-rodent Diversity	-0.189	0.555	-0.797	1	-0.561
Proportion EVEV Positive Pools	-0.109	-0.458	0.566	-0.561	1

Supplementary Figure 1. Model residuals relative cotton rat host use and EVEV infection rate versus longitude (left panels) and distance (right panels).

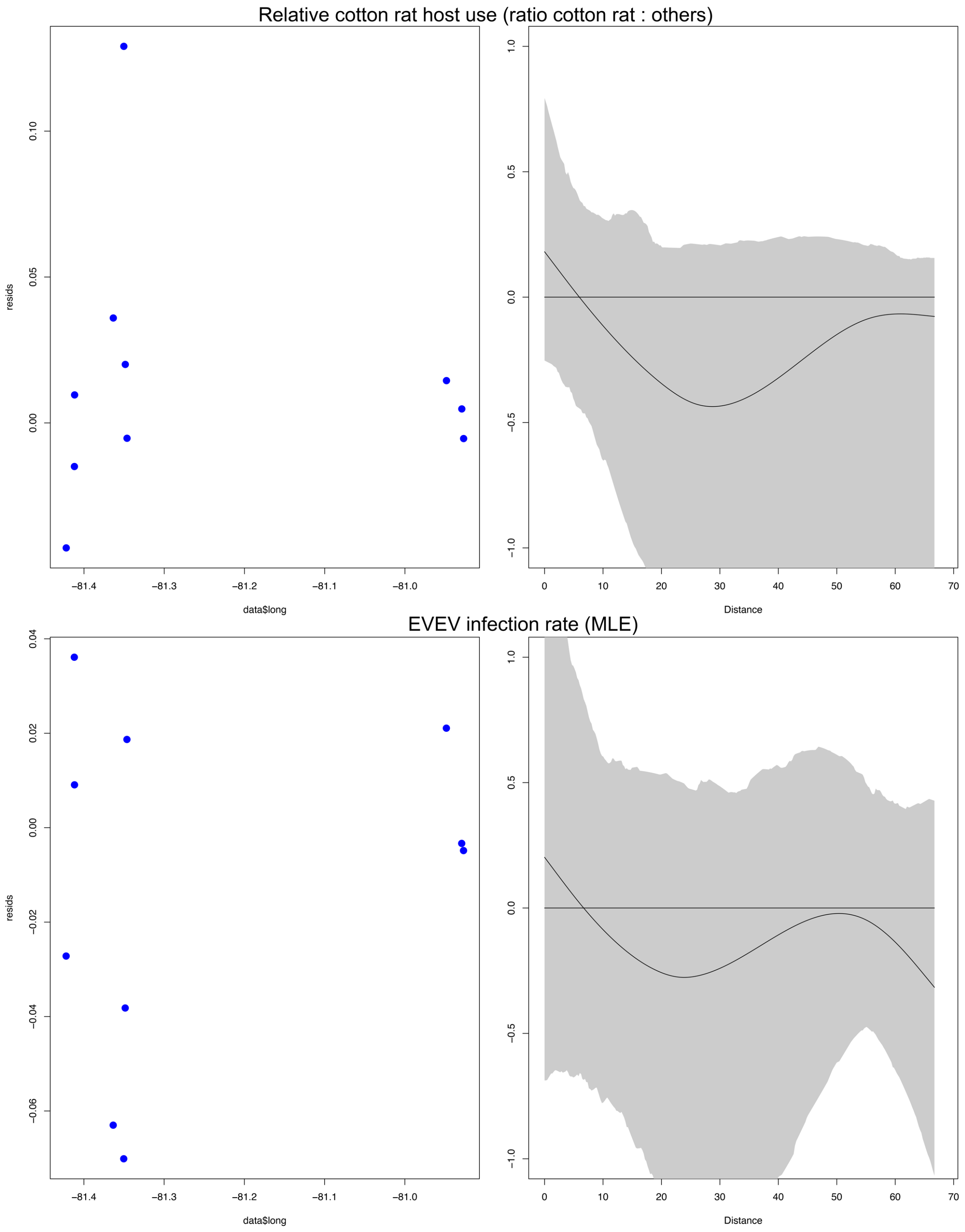

Supplementary Figure 2. Burmese python occurrence records used in species distribution models. Georeferenced points (yellow dots) were restricted to the years 2014 to 2017 and dowloaded from the Early Detection and Distribution Mapping Systems (EDDMapS) repository (https://www.eddmaps.org).

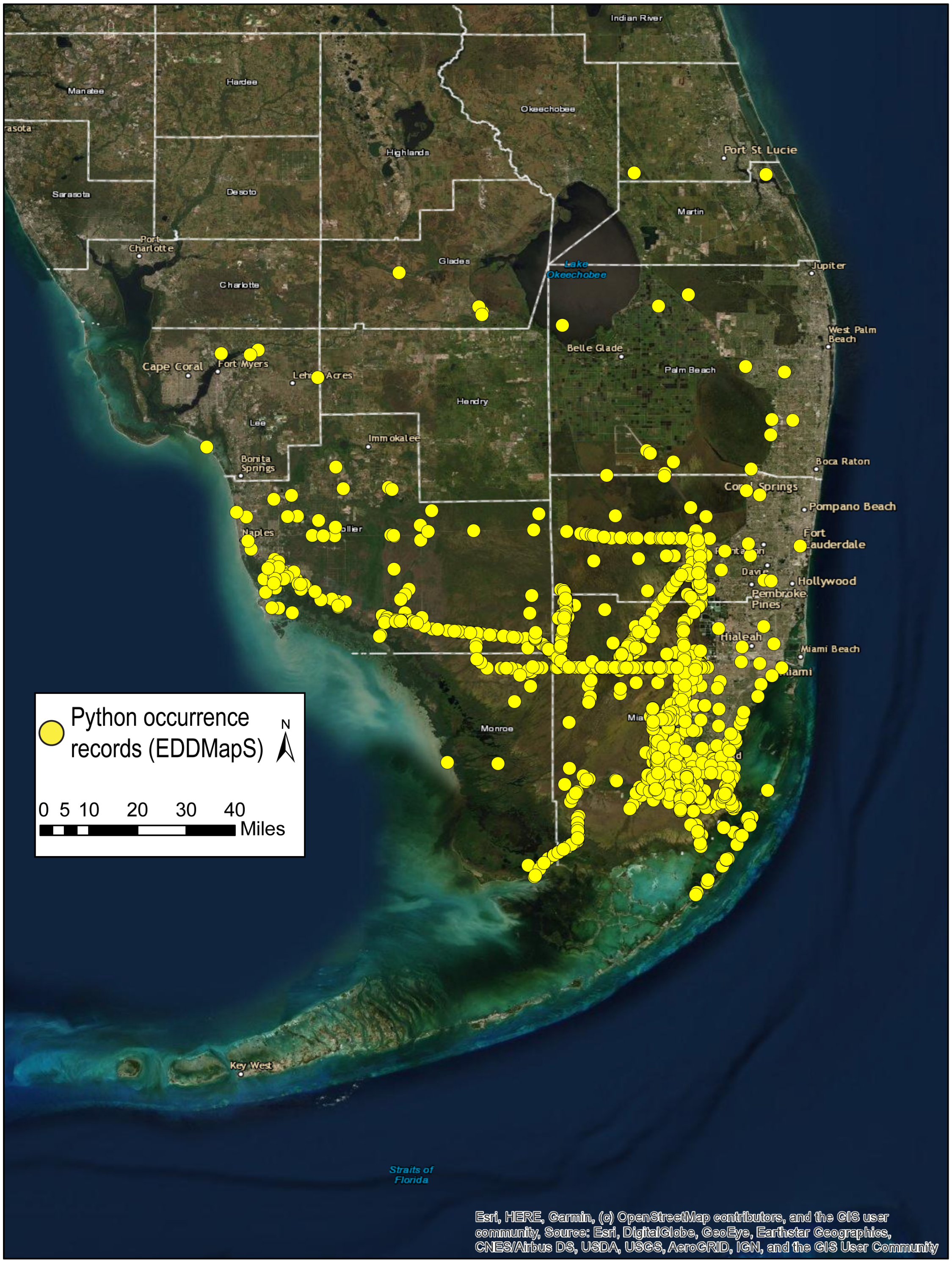

Supplementary Table 3. Sequences and combinations of primers used for conventional RT-PCR for Alphavirus screening and sequencing of EVEV E1 gene. Cycling temperatures for step 2 of the 35 cycle repeats of the cDNA amplification step are listed by primer set. Fluorochrome and Quencher for P2 were ATTO425 and BHQ1/LNA.

**Alphavirus screening**	**Primer Name**		**Sequence**
	F2A		ATGATGAARTCIGGIATGTTYYT
	R2A		ATYTTIACTTCCATGTTCATCCA
	R3A		ATYTTIACTTCCATRTTCARCCA
	R4A		ATYTTIACTTCCATGTTGACCCA
	P2		AT + GTT + GTC + GT + CIC + CIAT
**Sequencing of EVEV E1 gene**	**Primer Name**	**Genome Position (bp)**	**Sequence**
	Forward 9796	9796	GAGTCCTTGGACCACCTCTG
	Forward 10279	10279	AAATCAGAGGATTGCCTTGC
	Forward 10804	10804	GCCTTGTTTACCAGGGTGTC
	Reverse 10657	10657	CCTGAGTGTATGGCACATGG
	Reverse 11140	11140	TTTGGGCATGATACTGTGGA
	Reverse 11327	11327	CAATCGCCGCAAGTTCTATC
	**Set Name**	**Forward Primer**	**Reverse Primer**
	Set 2	Forward 9796	Reverse 10657
	Set 4	Forward 10279	Reverse 11140
	Set 6	Forward 10804	Reverse 11327
	Set 7	Forward 9796	Reverse 11327

Supplementary Table 4. Polymerase chain reaction primers used in host blood meal identification.

**Host Group**	**Primer Name**	**Sequence**	**Amplicon size (bp)**
Mammal / amphibian	L2513	5’-GCCTGTTTACCAAAAACATCAC-3’	300
	H2714	5’-CTCCATAGGGTCTTCTCGTCTT-3’	
Avian	L0	5’-GGACAAATATCATTCTGAGG-3’	220
	H1	5’-GGGTGGAATGGGATTTTGTC-3’	
Reptile	16L1	5’-CTGACCGTGCAAAGGTAGCGTAATCACT-3’	450
	H3056	5’-CTCCGGTCTGAACTCAGATCACGTAGG-3’	
