# Supplementary material for: Invasive Burmese pythons alter host use and virus infection in the vector of a zoonotic virus
Source: Commun Biol. 2021 Jun 28;4:804. doi: 10.1038/s42003-021-02347-z (PMC8239020; doi:10.1038/s42003-021-02347-z)
Supplement: Supplementary file 3 — Description of Additional Supplementary Files [file 42003_2021_2347_MOESM3_ESM.pdf]

Description of Additional Supplementary Files

File Name: Supplementary Data 1
Description: Host use of Culex cedecei from Florida, USA 2017. Vertebrate hosts of female mosquitoes determined by PCR and sequencing targeting conserved genes. Female mosquitoes were collected using artificial resting shelters (RT) and carbon dioxide-baited light traps (LT) at areas spanning a range of mammal communities and Burmese python abundances.

File Name: Supplementary Data 2
Description: Everglades virus infection in Culex cedecei from Florida, USA 2017. Pools of female mosquitoes were screened (mean pool size = 24.2 females) by RT-qPCR) assay targeting non-structural protein 4. Female mosquitoes were collected using artificial resting shelters and baited light traps at areas spanning a range of mammal communities and Burmese python abundances.
