# Supplementary material for: Invasive Burmese pythons alter host use and virus infection in the vector of a zoonotic virus
Source: Commun Biol. 2021 Jun 28;4:804. doi: 10.1038/s42003-021-02347-z (PMC8239020; doi:10.1038/s42003-021-02347-z)
Supplement: Supplementary file 6 — Reporting Summary [file 42003_2021_2347_MOESM6_ESM.pdf]

nature research

Corresponding author(s): Nathan Burkett-Cadena

Last updated by author(s): 03-16-2021

Reporting Summary

Nature Research wishes to improve the reproducibility of the work that we publish. This form provides structure for consistency and transparency in reporting. For further information on Nature Research policies, see our Editorial Policies and the Editorial Policy Checklist.

Statistics

For all statistical analyses, confirm that the following items are present in the figure legend, table legend, main text, or Methods section.

n/a	Confirmed	
☐	☒	The exact sample size (*n*) for each experimental group/condition, given as a discrete number and unit of measurement
☒	☐	A statement on whether measurements were taken from distinct samples or whether the same sample was measured repeatedly
☒	☐	The statistical test(s) used AND whether they are one- or two-sided<br>*Only common tests should be described solely by name; describe more complex techniques in the Methods section.*
☐	☒	A description of all covariates tested
☐	☒	A description of any assumptions or corrections, such as tests of normality and adjustment for multiple comparisons
☐	☒	A full description of the statistical parameters including central tendency (e.g. means) or other basic estimates (e.g. regression coefficient) AND variation (e.g. standard deviation) or associated estimates of uncertainty (e.g. confidence intervals)
☒	☐	For null hypothesis testing, the test statistic (e.g. *F*, *t*, *r*) with confidence intervals, effect sizes, degrees of freedom and *P* value noted<br>*Give P values as exact values whenever suitable.*
☒	☐	For Bayesian analysis, information on the choice of priors and Markov chain Monte Carlo settings
☒	☐	For hierarchical and complex designs, identification of the appropriate level for tests and full reporting of outcomes
☐	☒	Estimates of effect sizes (e.g. Cohen's *d*, Pearson's *r*), indicating how they were calculated

Our web collection on statistics for biologists contains articles on many of the points above.

Software and code

Policy information about availability of computer code

Data collection	To produce relative probability of Burmese python presence values across the region and at our study sites, we downloaded 1,165 georeferenced python observations collected during a four year period, prior to the year of the field study (2014 to 2017) from the Early Detection and Distribution Mapping Systems (EDDMapS) repository (https://www.eddmaps.org).
Data analysis	The species distribution model was generated using an ensemble modeling approach that combined model outputs from nine SDM algorithms, executed in the 'biomod2' package in R using default settings. 50. R Core Team R: A language and environment for statistical computing. R Foundation for Statistical Computing, Vienna, Austria. URL https://www.R-project.org/. (2019).

For manuscripts utilizing custom algorithms or software that are central to the research but not yet described in published literature, software must be made available to editors and reviewers. We strongly encourage code deposition in a community repository (e.g. GitHub). See the Nature Research guidelines for submitting code & software for further information.

Data

Policy information about availability of data

All manuscripts must include a data availability statement. This statement should provide the following information, where applicable:

- Accession codes, unique identifiers, or web links for publicly available datasets
- A list of figures that have associated raw data
- A description of any restrictions on data availability

All relevant data supporting the findings of this study are within the paper and its Supplementary Files. Any further data or information are available from the corresponding author upon reasonable request.

Field-specific reporting

Please select the one below that is the best fit for your research. If you are not sure, read the appropriate sections before making your selection.

☒ Life sciences ☐ Behavioural & social sciences ☐ Ecological, evolutionary & environmental sciences

For a reference copy of the document with all sections, see nature.com/documents/nr-reporting-summary-flat.pdf

Life sciences study design

All studies must disclose on these points even when the disclosure is negative.

Sample size	We conducted mosquito sampling on ten separate occasions in September and October 2017, with 3-6 sampling days per location. The numbers of mosquitoes captured was a function of the number of sampling events and the natural mosquito abundance at a given location. We quantified mammal communities at all twelve sites using cameras and live trapping. We sampled each site for three weeks (one week per month) with three digital trap cameras (Bushnell Trophy Camera, Overland Park, KS) placed in areas of potential mammal activity (i.e. trails). Additionally, at each site we placed 20 H. B. Sherman folding traps (3 x 3.5 x 9", H. B. Sherman Traps, Inc., Tallahassee, FL, USA) in two parallel 10-trap transects with 10 m spacing. We opened these traps for four consecutive nights every month for three months (12 days total). The numbers of mammals captured / detected was a function of the number of sampling events and the natural abundance at a given location.
Data exclusions	Locations with <4 blood meals (n=2) were excluded from statistical analyses.
Replication	The field techniques (mosquito and mammal sampling) were replicated across twelve independent sites. Because each site naturally varied in mammal community, mosquito density and Burmese python presence, duplication of results were not expected.
Randomization	A Pearson's correlation matrix identified the potential for multicollinearity between predictor variables, and we included only variables with r < ± 0.6 in the same candidate model.
Blinding	Blinding was not relevant to our study because data were collected from animals / field locations.

Reporting for specific materials, systems and methods

We require information from authors about some types of materials, experimental systems and methods used in many studies. Here, indicate whether each material, system or method listed is relevant to your study. If you are not sure if a list item applies to your research, read the appropriate section before selecting a response.

Materials & experimental systems

n/a	Involved in the study	
☒	☐	Antibodies
☒	☐	Eukaryotic cell lines
☒	☐	Palaeontology and archaeology
☐	☒	Animals and other organisms
☒	☐	Human research participants
☒	☐	Clinical data
☒	☐	Dual use research of concern

Methods

n/a	Involved in the study	
☒	☐	ChIP-seq
☒	☐	Flow cytometry
☒	☐	MRI-based neuroimaging

Animals and other organisms

Policy information about studies involving animals; ARRIVE guidelines recommended for reporting animal research

Laboratory animals	No laboratory animals were used.
Wild animals	Rodents (Hispnd cotton rat [Sigmodon hispidus], Cotton mouse [Peromyscus gossypinus], Black rat [Rattus rattus], Rice rat [Oryzomys palustris]) and shrews (undetermined species) were captured using Sherman folding traps (3 x 3.5 x 9", H. B. Sherman Traps, Inc., Tallahassee, FL, USA) in two parallel 10-trap transects with 10 m spacing. We opened these traps for four consecutive nights every month for three months (12 days total). We identified all rodent species and fitted each individual with an ear-tag (1005-1, National Band Co., Newport, KY, USA). All small mammals were released at point of capture. Images of wild animals were obtained with digital trap cameras (Bushnell Trophy Camera, Overland Park, KS) placed in areas of potential mammal activity (i.e. trails). Mammals were identified to species from the photographs (Armadillo [Dasypus novemcinctus], Mink [Neovion vison], Gray Squirrel [Sciurus carolinensis], Black Bear [Ursus americanus floridanus], White-tailed Deer [Odocoileus virginianus], Florida Panther [Puma concolor coryi], Marsh Rabbit [Sylvilagus palustris], Opossum [Didelphis viriginiana], Raccoon [Procyon lotor], Bobcat [Lynx rufus] and Coyote [Canis latrans].
Field-collected samples	After collection, mosquito samples were transported on dry ice, then stored at -80C until DNA or RNA extraction.

Ethics oversight	Our protocols were approved by the University of Florida Institution Animal Use and Care Committee (#201709906).

Note that full information on the approval of the study protocol must also be provided in the manuscript.
